# Identification and location of hot and cold spots of treated prevalence of depression in Catalonia (Spain)

**DOI:** 10.1186/1476-072X-11-36

**Published:** 2012-08-24

**Authors:** José A Salinas-Pérez, Carlos R García-Alonso, Cristina Molina-Parrilla, Esther Jordà-Sampietro, Luis Salvador-Carulla

**Affiliations:** 1 Universidad Loyola Andalucía, Business Administration Faculty, Sevilla, Córdoba, Spain; 2 Direcció General de Regulació, Planificació i Recursos Sanitaris, Departament de Salut, Generalitat de Catalunya, Barcelona, Spain; 3 Faculty of Health Sciences, University of Sydney, Sydney, Australia

**Keywords:** Spatial analysis, Hot spots, Cold spots, Mental health, Depression, Catalonia

## Abstract

**Background:**

Spatial analysis is a relevant set of tools for studying the geographical distribution of diseases, although its methods and techniques for analysis may yield very different results. A new hybrid approach has been applied to the spatial analysis of treated prevalence of depression in Catalonia (Spain) according to the following descriptive hypotheses: 1) spatial clusters of treated prevalence of depression (hot and cold spots) exist and, 2) these clusters are related to the administrative divisions of mental health care (catchment areas) in this region.

**Methods:**

In this ecological study, morbidity data per municipality have been extracted from the regional outpatient mental health database (CMBD-SMA) for the year 2009. The second level of analysis mapped small mental health catchment areas or groups of municipalities covered by a single mental health community centre. Spatial analysis has been performed using a Multi-Objective Evolutionary Algorithm (MOEA) which identified geographical clusters (hot spots and cold spots) of depression through the optimization of its treated prevalence. Catchment areas, where hot and cold spots are located, have been described by four domains: urbanicity, availability, accessibility and adequacy of provision of mental health care.

**Results:**

MOEA has identified 6 hot spots and 4 cold spots of depression in Catalonia. Our results show a clear spatial pattern where one cold spot contributed to define the exact location, shape and borders of three hot spots. Analysing the corresponding domain values for the identified hot and cold spots no common pattern has been detected.

**Conclusions:**

MOEA has effectively identified hot/cold spots of depression in Catalonia. However these hot/cold spots comprised municipalities from different catchment areas and we could not relate them to the administrative distribution of mental care in the region. By combining the analysis of hot/cold spots, a better statistical and operational-based visual representation of the geographical distribution is obtained. This technology may be incorporated into Decision Support Systems to enhance local evidence-informed policy in health system research.

## Background

Spatial epidemiology is aimed at identifying patterns in the geographical distribution of health data. It may detect irregularities such as spatial clusters of a particular disease [1,2], for example, where a specific disease has significant high or low prevalence [3]. Methods for the study of spatial clusters include global spatial autocorrelation, Local Indicators of Spatial Association (LISA), spatial regression, spatial scan statistics and Bayesian inference [4].

There are numerous examples of spatial data analysis performed on health variables, such as prevalence, incidence and mortality [5]. In mental health, for example, Bayesian models have been used to study the relationship between poverty and social isolation, and psychiatric admission rates in acute hospitals in small urban areas of London and New York [6]; the variation in the incidence of psychotic disorders in urban areas in Southeast London [7]; the relationship between depression and schizophrenia admission rates and socioeconomic characteristics in the counties of 14 States in the USA [8,9]; and the study of the correlation between mental retardation and clusters of developmental delay [10]. Spatial scan statistics have been used to detect clusters of mental disorders due to psychoactive substance use, and neurotic, stress-related, and somatoform disorders, and their relationship to poverty and neighbourhood social disorganization in Malmö (Sweden) [11]. LISA were applied to analyze spatial patterns of mental health in the slums of Dhaka (Bangladesh) [12]. In addition, a spatial regression model has been used to analyze spatial allocation in mental health expenditure in England [13].

However, the studies on spatial analysis show significant problems with respect to comparability, reproducibility and generalization since different methods and techniques produce different results [14,15]. We previously developed and tested [3] a Multi-Objective Evolutionary Algorithm (MOEA) that hybridised three LISA methods (Moran’s *I*, Geary’s *C* and Getis and Ord’s *G*) and Bayesian inference to detect schizophrenia hot spots (geographical clusters of spatial units –municipalities- with significantly high rates of selected indicators of a given disease) in Andalusia (Spain). Although this hybrid technique proved to be highly effective for this aim, there were problems when trying to precisely identify the location, shapes and boundaries of the spots, as also commonly occurs with other methods of spatial analysis [16,17].

This study has incorporated the identification of cold spots (geographical clusters of spatial units –municipalities- with significantly low rates of treated prevalence of a given disease) into the spatial analysis of the regional mental health system in Spain. The presence of both spatial clusters were analysed using the outpatient mental health database in Catalonia (Spain).

This paper aims to obtain a precise identification and geographical location of hot and cold spots of treated prevalence of depression and check if they have any spatial relationship with the administrative (catchment areas) divisions of mental health care in Catalonia in order to facilitate evidence to enable well-informed policy decisions. The related descriptive hypotheses are: 1) spatial clusters of treated prevalence of depression (hot and cold spots) exist and, 2) these clusters are related to the administrative divisions of mental health care (catchment areas) in Catalonia.

## Methods

### Design

This ecological study explores the geographical distribution of depression in 946 Catalonia municipalities (considered as our spatial units in this analysis) in 2009. Catalonia is a broad region in North Eastern Spain with 7.5 million inhabitants. It is one of the most developed Spanish regions with GDP 123.75 Purchasing Power Parity (European Union one = 100) [18]. Its public health system is universal with separate planning and provision and includes both public and private organisations under contract agreements with the public health system [19]. Mental health care in Catalonia is organized territorially in 74 small catchment areas coordinated by a reference Mental Health Community Centre (MHCC). These outpatient mental health centres follow a community care model, and are coordinated with primary care, specialized hospital care and intermediate care services.

We selected the municipalities as spatial units for precise geographical identification and location of hot and cold spots of treated prevalence of depression. These are the smallest areas where reliable statistical information can be found. Mental health catchment areas were selected as secondary units of analysis. Seven urban municipalities comprise more than one mental health area. All other mental health catchment areas include several municipalities.

### Database

Psychiatric cases assisted in the 74 Adult MHCC -catchment areas- in Catalonia are registered in the Minimum Data Set for Outpatient Mental Health Centres (CMBD-SMA) [20]. This study has used the 2009 database provided by the Catalonian Department of Health, safeguarding the privacy of the patients by using anonymous registers analysed at a municipality level to prevent geographical identification of individual cases. The database collects data from anonymous patients: gender, age, residence municipality, diagnosis, activity types, date of admission and discharge, etc. The variables used to calculate de treated prevalence were: sex, age, municipality of residence and main diagnosis (single episode, depressive disorder (F32) and recurrent-episode depressive disorder (F33) (ICD-10) [21]).

The CMBD-SMA 2009 database comprises information about 214,000 patients in total. A preliminary analysis removed 0.8% of them because the selected variables were incomplete or erroneous. Furthermore, 7 catchment areas did not provide complete information for that year. The final number of depressive patients analysed in this study was 24,580. The number of inhabitants (year 2009) in each municipality was obtained from the municipal census. Patient sex and age provided information to calculate the standard rates of treated prevalence of depression (per 1,000 population) through the direct method [22] that took into consideration the population of Catalonia.

Catchment areas in Catalonia have been described using four domains: urbanicity, service availability, accessibility to care and adequacy or appropriateness. These domains have been used in previous studies about the spatial distribution of mental illnesses prevalence. If hot/cold spots are spatially associated with specific catchment areas, it could be relevant to analyse if they are mainly rural or urban, if their accessibility is high or not and so on. The urbanicity level can be ‘predominantly urban’ when 85% of the inhabitants reside in municipalities whose density is greater than 150 inhabitants/km^2^, ‘significantly rural’ when this percentage is between 50% and 84%, and ‘predominantly rural’ when it is lower than 50% [23]. The accessibility to the MHCC of each catchment area was assessed using a standard Geographical Information System (GIS) in Catalonia (minutes by car to the corresponding MHCC from the less accessible zone of the catchment area) [24]. MHCC availability was measured by the rate of outpatient MHCC per 100,000 inhabitants. The adequacy of the provision of services in the mental health catchment areas was assessed by a group of PSICOST experts using information from the Mental Health Atlas of Catalonia [25]. Experts rated every catchment area by 7 levels of provision (very high, high, medium high, medium, medium low, low and very low). This rating was represented in semaphore scale and agreed with official ones from the Department of Health of Catalonia.

An exploratory spatial analysis on treated prevalence of depression in Catalonia was carried out to check whether its geographical distribution is distributed at random or not. Both global Moran’s *I* and Getis & Ord’s *G* were used [26,27].

### A Multi-Objective Evolutionary Algorithm (MOEA) applied to spatial data analysis

The full technical aspects of the MOEA model are described elsewhere [3,28]. MOEA are tools used to solve complex and usually non-linear multi-objective problems through optimization to achieve feasible and non-dominated efficient solutions [29]. The processes of optimization in MOEA are based on artificial intelligence techniques (evolutionary algorithms) and solutions (in our case, potential hot and cold spots) that are evaluated by means of different types of equations called fitness functions. These fitness functions assess the corresponding fitness degree of the solutions found in each run of the algorithm and they are designed by the objectives selected in the specific study (for example, the mean of treated prevalence of depression in a set of municipalities has to be maximized to identify spatial hot spots). The fitness value obtained represents the degree of agreement among the objectives selected to design the fitness function (improving one specific objective can lead to the worsening of another). MOEA improves solutions iteratively; in each run new and better solutions are obtained through classical genetic operators based on Nature: selection, mutation and crossover. Thus, the solution of the multi-objective problem is not unique, as there are many efficient solutions in response to the problem.

Our MOEA was designed to search for efficient solutions (potential hot and cold spots) by means of the optimization of three objectives that defined the fitness functions. MOEA analyses 100 sets of ‘*n*’ municipalities ( *n* = 10) identified by their standard codes (ie. [14004, 28097, 7009]). The initial group of 100 sets of 10 municipalities is selected at random and the improvement process starts. The standard genetic operators (selection, mutation, replication and elitism) are systematically used to improve the values of the fitness functions for each of the mentioned 100 sets of 10 municipalities (each set has their own values for the fitness functions). For example, mutation changes one municipality code in a specific set by other completely different (usually geographically close to the rest). This process stops when the values of the fitness functions for all the sets cannot be improved by the MOEA. In order to guarantee unexpected bias, the global process is repeated five times (five different initial groups of 100 sets of 10 municipalities). The objectives that structure the fitness functions were:

· Maximize (for hot spots) or minimize (for cold spots) the mean of the treated prevalence of depression (*P*) in a set of n municipalities ( *n* = 10). For hot spots: $\text{Max}\bar{P}$ and for cold spots: $\text{Min}\bar{P}$.

· Minimize the Standard Deviation (*SD*) of the treated prevalence of depression *SD*_P_ in the same set of n municipalities (*n* = 10). For both hot and cold spots: Min *SD*_*P*._

· Minimize the minimum distance *MinD* that links all the municipalities in the solution. For both hot and cold spots: Min *MinD*.

**Figure 1:**
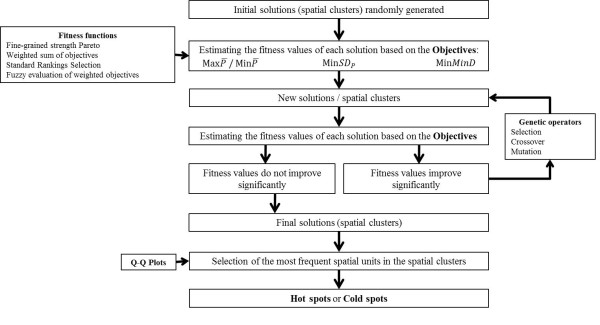
**Procedure for identifying hot spots and cold spots using MOEA.** ($\text{Max}\bar{P}$: maximize the average prevalence of depression; $\text{Min}\bar{P}$: minimize the average prevalence of depression; Min*SD*_*P*_: minimize the standard deviation of the depression prevalence; Min*MinD*: minimize de minimum distance between municipalities).

Four fitness functions were designed combining the three objectives for both hot spots:$\text{Max}\bar{P}$, Min*SD*_*P*._ and Min*MinD*; and for cold spots: $\text{Min}\bar{P}$, Min*SD*_*P*_ and Min*MinD*. These fitness functions were: Fine-Grained strength Pareto, weighted objectives, standard ranking selection and fuzzy evaluation of weighted objectives [3]. The procedure is summarized in Figure1. MOEA initially analyses 100 sets of *n* municipalities ( *n* = 10) –five times, but during the improvement process –genetic operators- the algorithm selects the best ones and, at the end, it gives an unpredictable –less than 100- number of *n* municipalities sets. The results are sets of municipalities where the mean of the treated prevalence of depression is high (potential hot spots) or low (potential cold spots), the standard deviation of their prevalence is low and, finally, the minimum distance that links all the n municipalities is low (therefore confirming the assumption that they are geographically close together).

Spatial units –municipalities- that appeared the most frequently, from a statistical point of view, in the potential hot and cold spots were selected as the final solution of the model: final hot and cold spots. This selection was performed using a standard procedure for identifying extreme values in a statistical distribution (Q-Q Plot method) [30]. The threshold values vary for each fitness function according to the calculated statistical frequency of the municipalities; the Q-Q Plot method selects the municipality from which the exponential and/or Pareto model provides a plausible statistical fit for the distribution of frequencies obtained. Spatial units that rarely appeared in the spots were not included in the final solution because the statistical analysis considered them to be spurious results. Hot and cold spots were finally mapped using the Geographical Information System (GIS) ArcGIS 9©.

In order to check the differences in the statistical distributions of treated prevalence of depression in the hot/cold spot and in the rest of Catalonia, Kruskal-Wallis’ one-way analysis of variance and Mann–Whitney *U* were used.

## Results and discussion

### Hot and cold spots of treated prevalence of depression

Spatial analysis searching for geographical patterns is of growing importance in epidemiology and in the evidence-informed paradigm which regards local data as a critical component for generating knowledge for planning and health policy. As stated by Lewin and colleagues, the evidence nearest to health decision-makers is that which informs about local conditions in their environment and is necessary to judge what decisions and actions must be taken in health policy [31]. It is also important to apply these techniques to study mental disorders such as depression, given its impact on the cost and burden of diseases [32,33] and the scarcity of prior information on spatial analysis in these conditions.

**Figure 2:**
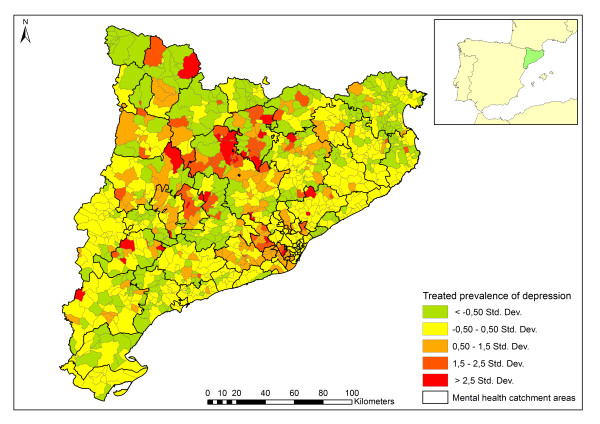
**Spatial distribution of the treated prevalence of depression (cases/1,000 inhabitants, 946 spatial units: municipalities) of Catalonia.** Intervals generated by the mean plus/minus a number of times multiplied by the standard deviation (Std. Dev.).

Treated prevalence of depression in Catalonia (year 2009) was 3.3 per 1,000 population. The standardized treated prevalence of depression per municipality is shown in Figure2. Five statistical classes based on standard deviation have been represented. There is a higher prevalence of depression located in the central northern region and in many disperse areas. Although the spatial distribution of the prevalence does not show a clear territorial pattern, it cannot be attributed to a random effect (Moran’s *I* = 0.19, *z* = 12.5, α ≤ 0.01; Getis & Ord’s *G* = 0, *z* = 12.05, α ≤ 0.01).

**Figure 3:**
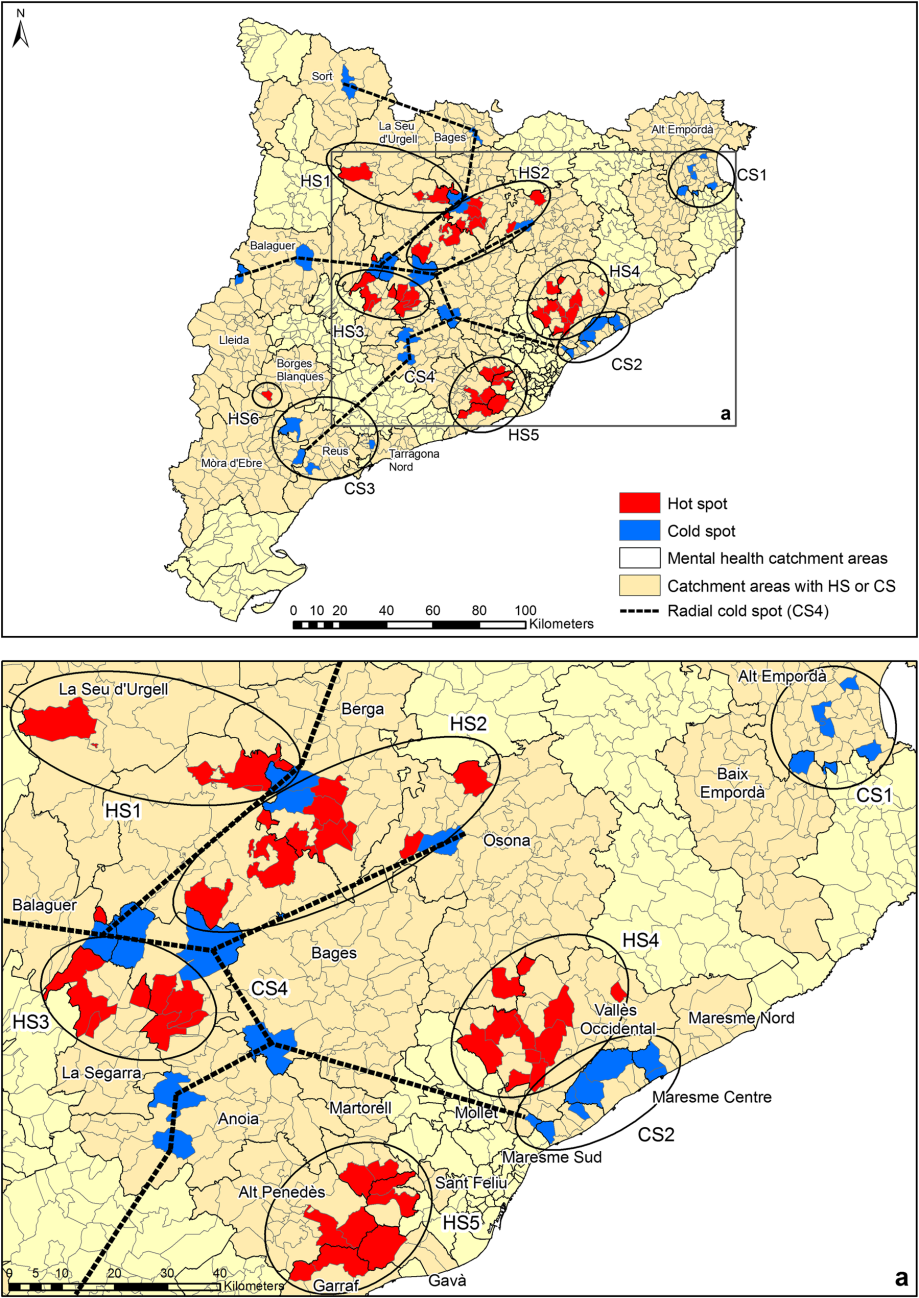
Spatial distribution of hot spots and cold spots of depression treated prevalence.

Hot spots and cold spots of treated prevalence of depression are represented in Figure3. Five hot spots (HS1-5) and one isolated municipality (HS6) have been found. Additionally, three cold spots (CS1-3) plus a radial cluster of several municipalities (CS4) have been identified by the model. The radial cluster CS4 delimits the hot spots HS1, HS2 and HS3. Two well-defined hot spots (HS4 and HS5) are clearly identified in Figure3. One of them (HS4) is adjacent to CS2, while the other cold spots (CS1 and CS3) are completely isolated.

Table1 shows the basic statistics of the treated prevalence of depression in the hot/cold spots identified, as well as the catchment areas. There are two hot spots with a mean prevalence over 10 per 1,000 inhabitants (HS2 and HS3). It is important to note that in these hot spots the standard deviations of prevalence are also very high as both hot spot group municipalities have very different rates of depression. On the other hand, HS1, HS4 and HS5 show lower standard deviations. The mean and standard deviation of some cold spots are zero (CS1 and CS3) or very low (CS2 and CS4). In these cases, our methodology was seeking geographical zones with very low prevalence, so the algorithm was successful in identifying cold spots.

**Table 1:** Basic statistics, geographical location and characteristics of the hot/cold spots of treated prevalence of depression in Catalonia (2009) (946 municipalities)

**SU**	**Number of municipalities and basic statistics in HS/CS**	**Location of HS/CS (Catchment area name)**	**Urbanicity of catchment areas where HS/CS are located (Type)**	**Availability of MHCC [**[25]**] (Rate per 100,000 pop.)**	**Road accessibility to a MHCC in catchment areas in minutes [**[24]**]**	**Adequacy of provision of mental health services [**[25]**]**
HS1	*N: 3 m; Mean: 9.9; Median: 8.3; St. Dev: 3.4*	Bages	Significantly rural	0.6	0 - 60	Very high
		Seu d’Urgell	Significantly rural	5.6	0 - >60	Low
HS2	*N: 8 m; Mean: 12.0; Median: 9.0;St. Dev: 10.1*	Bages	Significantly rural	0.6	0 - 60	Very high
		Berga	Significantly rural	3.0	0 - 45	High
		Osona	Predominantly urban	0.8	0 - 45	Very high
HS3	*N: 7 m; Mean: 11.6; Median: 9.6;St. Dev: 6.6*	Anoia	Significantly rural	1.1	0 - 60	Low
		La Segarra	Significantly rural	6.0	0 - 45	Medium
HS4	*N: 11 m; Mean: 4.6; Median: 3.3;St. Dev: 3.2*	Vallès Oriental	Predominantly urban	0.4	0 - 45	Very high
		Osona	Predominantly urban	0.8	0 - 45	Very high
HS5	*N: 9 m; Mean: 5.2; Median: 5.4;St. Dev: 2*	Alt Penedès	Significantly rural	1.3	0 - 45	High
		Garraf	Predominantly urban	0.8	0 - 30	High
		Gavà	Predominantly urban	1.1	0 - 15	High
		Martorell	Predominantly urban	0.8	0 - 30	Very high
		Sant Feliu	Predominantly urban	1.1	0 - 30	Medium
HS6	*N: 1 m; P: 13.9*	Borges Blanques	Predominantly rural	6.0	0 - 45	Medium
No HS	*N: 908 m; Mean: 2.3; Median: 1.9; St. Dev: 2.4*	-	-	-	-	-
CS1	*N: 6 m; Mean: 0; Median: 0; St. Dev: 0*	Alt Empordà	Significantly rural	0.9	0 - 60	Medium
		Gironès	Significantly rural	0.6	0 - 30	Very high
CS2	*N: 8 m; Mean: 1.1; Median: 1; St. Dev: 0.9*	Maresme Nord	Predominantly urban	0.9	0 - 45	Very high
		Maresme Centre	Predominantly urban	0.8	0 - 30	Very high
		Maresme Sud	Predominantly urban	1.1	0 - 15	Medium.
		Mollet	Predominantly urban	1.2	0 - 15	High
CS3	*N: 4 m; Mean: 0; Median: 0; St. Dev: 0*	Mòra d’Ebre	Significantly rural	2.8	0 - 45	Medium low
		Reus	Predominantly urban	0.6	0 - 45	Low
		Tarragona Nord	Predominantly urban	0.6	0 - 30	Medium low
CS4	*N: 13 m; Mean: 0.2; Median: 0; St. Dev: 0.8*	Anoia	Significantly rural	1.1	0 - 60	Low
		Bages	Significantly rural	0.6	0 - 60	Very high
		Balaguer	Predominantly rural	3.2	0 - >60	Medium
		Berga	Significantly rural	3.0	0 - 45	High
		Lleida	Significantly rural	0.6	0 - 45	High
		Osona	Predominantly urban	0.8	0 - 45	Very high
		Sort	Predominantly rural	11.8	0 - >60	Low
No CS	*N: 915 m; Mean: 2.6; Median: 2.0; St. Dev: 2.9*	-	-	-	-	-
Catalonia	*N: 946 m; Mean: 2.6; Median: 2.0; St. Dev: 2.9*	-	-	1.2	-	-

CS, cold spot; HS, hot spot; m, municipalities; MHCC, mental health community centre; No HS, Municipalities not included in hot spots; No CS, municipalities not included in cold spots; SU, spatial units.

Some spatial units with small populations –numbers of inhabitants– have been included in hot/cold spots. Treated prevalence might show a high longitudinal variation in areas with this characteristic where a variation of a few patients can greatly influence the overall treated prevalence. This behaviour might be due to a random variation in depression patients throughout the time span. MOEA searches clusters of close spatial units in the space, so the appearance of significantly high or low treated prevalence cannot be considered to be due to random effects and shows potential areas of interest for decision makers.

Statistical tests indicate that there are significant differences between hot and cold spots in the distribution of treated prevalence of depression in comparison with the rest of the municipalities in Catalonia (α ≤ 0.05). Hot/cold spots can be considered independent groups of spatial units, with different geographical location and rates of treated prevalence of depression.

### Catchment areas and hot/cold spots

The hot/cold spots of treated prevalence of depression in Catalonia are located in 25 of the 74 mental health catchment areas. The existence of hot/cold spots could not be attributed to the characteristics of the administrative division of mental health care in Catalonia as hot/cold spots could not be assigned to individual catchment areas. The spots include municipalities within different catchment areas. Therefore, the existence of hot/cold-spots cannot be attributable to a variation in clinical practice in specific MHCC.

On the other hand, municipalities in hot/cold spots inherit the main characteristics of the catchment areas in which they are included: urbanicity, availability, accessibility and adequacy. These domains are shown in Table1.

Hot spots and cold spots are mainly located in the central-northern and eastern regions of Catalonia except for CS3 in the south. The size of the whole region does not suggest any relationship with the geographical characteristics of the territory because the affected catchment areas are urban and rural, industrial and agriculture-based, etc. (Table1). HS1, HS2 and HS3 are located in mainly rural catchment areas although HS4 and HS5 have been identified in urban areas. On the other hand, CS1 and CS4 are located in predominantly rural areas while CS2 and CS3 are in urban ones. Treated prevalence of depression in Catalonia cannot easily be associated with urbanicity as has been claimed in other studies [34].

It has been stated that a long distance to a mental health specialist may reduce the numbers of visits of rural patients with depression [35]. There is no clear relationship between accessibility to MHCC and the location of hot/cold spots of treated prevalence of depression in Catalonia. According to Olivet et al. (2008), HS3, HS4, HS5, CS1 and CS2 are located in areas less than 30 minutes of travel time away. HS1, HS5, CS3 and CS4 are over 30 minutes away.

Areas with very low or very high accessibility to mental health services could be related to changes in the rates of treated prevalence for several reasons [36]. Perhaps MHCC could have an overload of cases due to the lack of other intermediate services. On the other hand, less availability of services may generate less demand and therefore a lower treated prevalence. However, the door-keeper effect of MHCC in a system organised by sectors and the lack of very-low adequacy areas rules out this possibility. On the other hand, previous studies have shown a relation between higher service utilisation and higher availability and provision. However, better accessibility may generate more cases and service use and an increase in cases attended in MHCC due to there being less diversity of services [37].

The availability of Adult MHCC per population of 100,000 in catchment areas with hot spots or cold spots is different, as seen in Table1. The adequacy of the provision of all specialized mental health services is high or very high in two-thirds of the catchment areas with hot spots while 13.3% of the areas have low provision. HS2, HS4 and HS5 have the highest adequacy while the lowest is in HS3. Half of the catchment areas with cold spots have high or very high provision while 31.3% are low or medium low. The cold spots with the best provision are CS1 and CS2, while CS3 is the lowest and CS4 shows a great degree of variation.

### Limitations of the study

This study is not aimed at identifying the causes of hot/cold spots. It is not possible to infer individual level relationship from relationship observed at the aggregate level due to the ecological fallacy [38,39]. The analysis of the Catalonia Health Survey indicates that the local burden of depression is associated with a low educational level and living alone [40]. The ESEMeD project shows the relationship between the prevalence of depression and sex, unemployment, civil status and disabilities [34].

On the other hand, the modifiable spatial unit problem is an additional difficulty in spatial analysis [1]. This problem refers to the variation in interpretations of statistics and results due to the size of the geographical area where individual data have been aggregated. The impact of both problems has been reduced in our study using the municipality scale. This aggregate unit is the best spatial unit available that does not compromise individual identification and confidentiality.

The spatial units located at the borders of the region are an important constraint for spatial analysis because the values of their neighbouring areas located in other regions or countries are not known. If these values were available, additional hot/cold spots could appear in the territory. However, this problem is reduced in Catalonia due to its geographical characteristics (a long seacoast) and the organisation of care by defined sectors.

This study uses specialised health databases; this information should be completed with the analysis of depression treated in primary care. Unfortunately primary care databases do not cover the whole territory of Catalonia and only partial information is available on mental disorders treated in the primary care system [41]. Furthermore, the specialised care registries are not complete in 7 out of the 74 mental health catchment areas.

## Conclusions

Hot spots and cold spots have previously been identified in a number of studies [42,43]. However, to our knowledge this is the first analysis that combines the optimization of basic statistics (mean and standard deviation) and the geographical location of hot and cold spots in one single procedure based on a hybrid model. A number of cold spots delimits different hot spots which would have been regarded as a single cluster otherwise. This analysis has also identified radial cluster patterns of cold spots which have not previously been described and which improve the identification of hot spots. It is especially interesting because it allows the identification of hot spots that could be considered to be one and the same due to their proximity, though each of them could be generated by different factors.

The relationship between hot and cold spots may have appeared in previous studies although their tentative relationship and meaning were not described [43]. In order to better understand the spatial distribution of a disease in the territory, hot and cold spots should be described together.

The location of both hot/cold spots may require specific actions including flexible health programs, plans and priority settings [44]. The visual representation of the results on maps can be a relevant component of the Knowledge Discovery Data applied to Health System research. It facilitates the elicitation of implicit expert knowledge to better understand complex information using, for example, Expert-based Cooperative Analysis (EbCA) [45].

Future studies may include the combined analysis of different databases (e.g. in primary and in tertiary care), the probing of the relationship between hot/cold spots in other diagnosis and territories, and the relationships between spatial clusters of treated prevalence of depression and the characteristics of mental health catchment areas and socioeconomic indicators through regression methods and ordinal classification.

## Abbreviations

CS: Cold spot; EbCA: Expert-based Cooperative Analysis; GIS: Geographic Information System; HS: Hot spot; ICD-10: International Statistical Classification of Diseases and Related Health Problems. 10th Revision; LISA: Local Indicators of Spatial Association; MHCC: Mental Health Community Centre; CMBD-SMA: Minimum Data Set for Outpatient Mental Health Centres; MOEA: Multi-Objective Evolutionary Algorithm.

## Competing interests

The authors declare that they do not have competing interests with regard to this manuscript.

## Authors’ contributions

CRGA and LSC designed the tool for the spatial analysis, JASP performed the data mining, data analysis and maps, and CMP and EJS analyzed and interpreted the results. All authors contributed to the writing of the manuscript. All authors read and approved the final manuscript.

## Authors’ information

for the GEOSCAT Group

Antoni Serrano, Ana Fernández, Teresa Marfull, Miriam Poole, Mencía Ruiz, María Luisa Rodero, Javier Álvarez, Josep María Haro, Esther Rovira, Josep Fusté, Cristina Romero and Bibiana Prat.

## References

[B1] Elliott P Wartenberg D Spatial epidemiology: current approaches and future challenges Environ Health Perspect 2004 112 998 1006 10.1289/ehp.6735 15198920 PMC1247193

[B2] Ward M Sui DZ Geospatial Technologies and Homeland Security Spatial Epidemiology: Where Have We Come in 150 Years? Volume 94 2008 Springer, Dordrecht 257 282

[B3] García-Alonso CR Salvador-Carulla L Negrín-Hernández MA Moreno-Küstner B Development of a new spatial analysis tool in mental health: identification of highly autocorrelated areas (hot-spots) of schizophrenia using a Multiobjective Evolutionary Algorithm model (MOEA/HS) Epidemiol Psichiatr Soc 2010 19 302 313 21322504

[B4] Auchincloss AH Gebreab SY Mair C Diez Roux AV A Review of Spatial Methods in Epidemiology, 2000–2010 Annu Rev Public Health 2012 33 107 122 10.1146/annurev-publhealth-031811-124655 22429160 PMC3638991

[B5] Bithell JF A classification of disease mapping methods Stat Med 2000 19 2203 2215 10.1002/1097-0258(20000915/30)19:17/18<2203::AID-SIM564>3.0.CO;2-U 10960848

[B6] Curtis S Copeland A Fagg J Congdon P Almog M Fitzpatrick J The ecological relationship between deprivation, social isolation and rates of hospital admission for acute psychiatric care: a comparison of London and New York City Health Place 2006 12 19 37 10.1016/j.healthplace.2004.07.002 16243678

[B7] Kirkbride JB Fearon P Morgan C Dazzan P Morgan K Murray RM Jones PB Neighbourhood variation in the incidence of psychotic disorders in Southeast London Soc Psychiatry Psychiatr Epidemiol 2007 42 438 445 10.1007/s00127-007-0193-0 17473901

[B8] Fortney JC Rushton G Wood S Zhang L Xu S Dong F Rost K Community-Level Risk Factors for Depression Hospitalizations Adm Policy Ment Health 2007 34 343 352 10.1007/s10488-007-0117-z 17294123

[B9] Fortney JC Xu S Dong F Community-Level Correlates of Hospitalizations for Persons With Schizophrenia Psychiatr Serv 2009 60 772 778 10.1176/appi.ps.60.6.772 19487346

[B10] Zhen H McDermott S Lawson A Aelion M Are clusters of mental retardation correlated with clusters of developmental delay? Geospat Health 2009 4 17 26 19908187 10.4081/gh.2009.207 PMC4648356

[B11] Chaix B Leyland AH Sabel CE Chauvin P Råstam L Kristersson H Merlo J Spatial clustering of mental disorders and associated characteristics of the neighbourhood context in Malmö, Sweden, in 2001 J Epidemiol Community Health 2006 60 427 435 10.1136/jech.2005.040360 16614334 PMC2563968

[B12] Gruebner O Khan MMH Lautenbach S Muller D Kramer A Lakes T Hostert P A spatial epidemiological analysis of self-rated mental health in the slums of Dhaka Int J Health Geogr 2011 10 36 10.1186/1476-072X-10-36 21599932 PMC3123168

[B13] Moscone F Knapp M Tosetti E Mental health expenditure in England: A spatial panel approach J Health Econ 2007 26 842 864 10.1016/j.jhealeco.2006.12.008 17296239

[B14] Moreno B García-Alonso CR Negrín Hernández M Torres-González F Salvador-Carulla L Spatial analysis to identify hotspots of prevalence of schizophrenia Soc Psychiatry Psychiatr Epidemiol 2008 43 782 791 10.1007/s00127-008-0368-3 18500483

[B15] Torabi M Rosychuk RJ An examination of five spatial disease clustering methodologies for the identification of childhood cancer clusters in Alberta, Canada Spat Spatiotemporal Epidemiol 2011 2 321 330 10.1016/j.sste.2011.10.003 22748230

[B16] Jacquez GM Kaufmann A Goovaerts P Boundaries, links and clusters: a new paradigm in spatial analysis? Environ Ecol Stat 2008 15 403 419 10.1007/s10651-007-0066-4 19023453 PMC2435220

[B17] Cançado AL Duarte AR Duczmal LH Ferreira SJ Fonseca CM Gontijo EC Penalized likelihood and multi-objective spatial scans for the detection and inference of irregular clusters Int J Health Geogr 2010 9 55 10.1186/1476-072X-9-55 21034451 PMC2990730

[B18] Eurostat Eurostat regional yearbook 2010 2010 Publications Office of the European Union, Luxembourg

[B19] Salvador-Carulla L Costa-Font J Cabases J McDaid D Alonso J Evaluating mental health care and policy in Spain J Ment Health Policy Econ 2010 13 73 86 20919594

[B20] Health and Social Security Department Notification Handbook of the Register of the Minimum Basic Data Set: Outpatient Mental Health Centers 2003 Catalonian Health Service, Barcelona

[B21] World Health Organization International Statistical Classification of Diseases and Related Health Problems 2011 2010 World Health Organization, Geneva Volume 2 Instruction manual

[B22] Rezaeian M Dunn G St Leger S Appleby L Geographical epidemiology, spatial analysis and geographical information systems: a multidisciplinary glossary J Epidemiol Community Health 2007 61 98 102 10.1136/jech.2005.043117 17234866 PMC2465628

[B23] OECD Creating rural indicators for shaping territorial policy 1994 Organisation for Economic Co-operation and Development, Paris

[B24] Olivet M Aloy J Prat E Pons X Health services provision and geographic accessibility Med Clin (Barc) 2008 131 Suppl 4 16 22 19195473 10.1016/s0025-7753(08)76470-4

[B25] GEOSCAT Group Integral Map of Mental Health Resources of Catalonia Department of Health of Catalonia, Barcelona In press

[B26] Anselin L Local indicators of spatial association-LISA Geogr Anal 1995 27 93 115

[B27] Ord JK Getis A Local spatial autocorrelation statistics: distributional issues and an application Geogr Anal 1995 27 186 306

[B28] García-Alonso CR Pérez-Naranjo LM Fernández-Caballero JC Multiobjective evolutionary algorithms to identify highly autocorrelated areas: the case of spatial distribution in financially compromised farms Ann Oper Res 2011 1 16

[B29] Coello-Coello C Lamont G Van Veldhuizen D Evolutionary algorithms for solving multi-objective problems 2007 Springer, New York

[B30] Beirlant J Goegebeur Y Teugels J Segers J Statistics of extremes: theory and applications 2004 Wiley, Chichester, West Sussex

[B31] Lewin S Oxman AD Lavis JN Fretheim A Garcia Marti S Munabi-Babigumira S SUPPORT tools for evidence-informed policymaking in health 11: Finding and using evidence about local conditions Health Res Policy Syst 2009 7 Suppl 1 S11 10.1186/1478-4505-7-S1-S11 20018101 PMC3271822

[B32] Gustavsson A Svensson M Jacobi F Allgulander C Alonso J Beghi E Dodel R Ekman M Faravelli C Fratiglioni L Gannon B Jones DH Jennum P Jordanova A Jönsson L Karampampa K Knapp M Kobelt G Kurth T Lieb R Linde M Ljungcrantz C Maercker A Melin B Moscarelli M Musayev A Norwood F Preisig M Pugliatti M Rehm J Salvador-Carulla L Schlehofer B Simon R Steinhausen H-C Stovner LJ Vallat J-M den Bergh PV van Os J Vos P Xu W Wittchen HU Jönsson B Olesen J Cost of disorders of the brain in Europe 2010 Eur Neuropsychopharmacol 2011 21 718 779 10.1016/j.euroneuro.2011.08.008 21924589

[B33] Wittchen HU Jacobi F Rehm J Gustavsson A Svensson M Jönsson B Olesen J Allgulander C Alonso J Faravelli C Fratiglioni L Jennum P Lieb R Maercker A van Os J Preisig M Salvador-Carulla L Simon R Steinhausen H-C The size and burden of mental disorders and other disorders of the brain in Europe 2010 Eur Neuropsychopharmacol 2011 21 655 679 10.1016/j.euroneuro.2011.07.018 21896369

[B34] Gabilondo A Rojas-Farreras S Vilagut G Haro JM Fernández A Pinto-Meza A Alonso J Epidemiology of major depressive episode in a southern European country: results from the ESEMeD-Spain project J Affect Disord 2010 120 76 85 10.1016/j.jad.2009.04.016 19428121 PMC3756284

[B35] Fortney JC Rost K Zhang M Warren J The impact of geographic accessibility on the intensity and quality of depression treatment Med Care 1999 37 884 893 10.1097/00005650-199909000-00005 10493467

[B36] Marín I Briones E Variability and clinic management. Concerning the use of the atlas for clinical Ulysses to overcome cyclop’s vision Atlas of Variations in Medical Practice 2007 2 139 141

[B37] Gittelsohn A Powe NR Small area variations in health care delivery in Maryland Health Serv Res 1995 30 295 317 7782218 PMC1070065

[B38] Macintyre S Ellaway A Cummins S Place effects on health: how can we conceptualise, operationalise and measure them? Soc Sci Med 2002 55 125 139 10.1016/S0277-9536(01)00214-3 12137182

[B39] Ocaña-Riola R Common errors in disease mapping Geospat Health 2010 4 139 154 20503184 10.4081/gh.2010.196

[B40] Sabes-Figuera R Knapp M Bendeck M Mompart-Penina A Salvador-Carulla L The local burden of emotional disorders. An analysis based on a large health survey in Catalonia (Spain) Gac Sanit 2012 26 24 29 10.1016/j.gaceta.2011.05.019 22078690

[B41] Aragonès E Salvador-Carulla L López-Muntaner J Ferrer M Piñol JL Registered prevalence of borderline personality disorder in primary care databases Gac Sanit In press 10.1016/j.gaceta.2011.12.006 22402239

[B42] Cheng C-L Chen Y-C Liu T-M Kao-Yang Y-H Using Spatial Analysis to Demonstrate the Heterogeneity of the Cardiovascular Drug-Prescribing Pattern in Taiwan BMC Publ Health 2011 11 380 10.1186/1471-2458-11-380 PMC3125367 21609462

[B43] Sridharan S Koschinsky J Walker JJ Does context matter for the relationship between deprivation and all-cause mortality? The West vs. the rest of Scotland Int J Health Geogr 2011 10 33 10.1186/1476-072X-10-33 21569408 PMC3103414

[B44] Koschinsky J The case for spatial analysis in evaluation to reduce health inequities Eval Program Plann In press 10.1016/j.evalprogplan.2012.03.004 22469340

[B45] Gibert K García-Alonso CR Salvador-Carulla L Integrating clinicians, knowledge and data: expert-based cooperative analysis in healthcare decision support Health Res Policy Syst 2010 8 28 10.1186/1478-4505-8-28 20920289 PMC2958926

